# Learning From Errors: Exploring the Effectiveness of Enhanced Error Messages in Learning to Program

**DOI:** 10.3389/fpsyg.2021.768962

**Published:** 2021-11-30

**Authors:** Zihe Zhou, Shijuan Wang, Yizhou Qian

**Affiliations:** ^1^Faculty of Artificial Intelligence in Education, Central China Normal University, Wuhan, China; ^2^Department of Educational Technology, Jiangnan University, Wuxi, China

**Keywords:** introductory programming, learning from errors, enhanced programming error messages, automated assessment tools, productive failures

## Abstract

Error messages provided by the programming environments are often cryptic and confusing to learners. This study explored the effectiveness of enhanced programming error messages (EPEMs) in a Python-based introductory programming course. Participants were two groups of middle school students. The control group only received raw programming error messages (RPEMs) and had 35 students. The treatment group received EPEMs and had 33 students. During the class, students used an automated assessment tool called Mulberry to practice their programming skill. Mulberry automatically collected all the solutions students submitted when solving programming problems. Data analysis was based on 6339 student solutions collected by Mulberry. Our results showed that EPEMs did not help to reduce student errors or improve students’ performance in debugging. The ineffectiveness of EPEMs may result from reasons such as the inaccuracy of the interpreter’s error messages or students not reading the EPEMs. However, the viewpoint of productive failure may provide a better explanation of the ineffectiveness of EPEMs. The failures in coding and difficulties in debugging can be resources for learning. We recommend that researchers reconsider the role of errors in code and investigate whether and how failures and debugging contribute to the learning of programming.

## Introduction

With the development of computing technologies, many countries have included computer science (CS) courses into K-12 curriculum (Webb et al., 2017). Programming is an essential part of CS education, but novices usually face a variety of difficulties when learning to program, one of which is debugging code (Qian and Lehman, 2017). When students make mistakes in their code, they receive error messages provided by the programming environments. Although these error messages contain detailed information about the errors in code, they are notoriously cryptic and confusing to novice programmers (Becker et al., 2019). Thus, students may not be able to utilize the information in the error messages to rectify their erroneous programs. Moreover, error messages provided by compilers and interpreters are not always accurate (McCall and Kölling, 2019). For instance, in Java programming, the same mistake (e.g., missing a semicolon) may result in different error messages when the syntactical contexts vary.

Many efforts have been made to help students better understand programming error messages and develop their debugging skills. One widely used approach is to develop automated assessment tools providing elaborated feedback when students make errors in their code (Pettit and Prather, 2017). Automated assessment tools (AATs) are popular in programming courses as they can automatically evaluate the correctness of students’ programs (Douce et al., 2005; Ihantola et al., 2010; Prather et al., 2018). Many researchers have designed and developed feedback components for AATs to help students understand errors in their code by providing enhanced error messages or other elaborated feedback (Denny et al., 2014; Becker et al., 2016; Keuning et al., 2018). While early research on error message enhancement started in 1960s, its effectiveness is still inconclusive (Pettit et al., 2017; Becker et al., 2019). This study implemented a data-driven approach to develop enhanced programming error messages for learners and examined the effectiveness of the enhancement on students’ performance in introductory programming.

### Related Work

Previous studies have used different terms to describe students’ errors in code, such as mistakes (Brown and Altadmri, 2017), syntax errors (Denny et al., 2012), compiler errors (Pettit et al., 2017), novice programmer errors (McCall and Kölling, 2019), and others (see Becker et al., 2019). However, these terms might be appropriate in certain contexts, but not in all research settings. For instance, compiler errors only exist in compiled programming languages (e.g., Java). It is not proper to use the term “compiler errors” to describe errors in an interpreted programming language such as Python (Kohn, 2019). In addition to syntax errors, which constitute one category of errors in programming, students also make semantic and logic errors (McCall and Kölling, 2014; Qian and Lehman, 2020).

After comprehensively reviewing relevant studies and analyzing the architecture of compilers and interpreters, Becker et al. (2019) suggested using the term “programming errors” to describe students’ errors in code. According to Becker et al. (2019), programming errors fall into two major categories: language specification errors and program specification errors. Language specification errors occur when the program violates the requirements of the programming language. Therefore, they can be detected by the compiler or the interpreter (e.g., compiler errors in Java and syntax errors in Python). When a well-formed program “does not behave according to its own specification of correctness” (Becker et al., 2019, p. 182), it contains program specification errors (e.g., logic errors). For example, consider a program designed to judge whether a number is prime or not that identifies (incorrectly) the number 4 as a prime number. This program has program specification errors, although it may not receive any error messages from the compiler or the interpreter.

Programming error messages are the messages provided by the programming environment to the user presenting the errors in the program. Most of the current research on this topic focuses on error messages to language specification errors (LSEs), because such errors messages are notoriously cryptic and confusing to novices (Becker et al., 2019). Prior research has used various negative words to describe error messages of compilers and interpreters since 1960s, including inadequate (Moulton and Muller, 1967; Brown, 1983), frustrating (Flowers et al., 2004; Becker et al., 2018), and undecipherable (Traver, 2010). As the standard error messages of programming environments are designed for experts instead of novices, it is not surprising that students find them confusing and difficult to understand (Nienaltowski et al., 2008; Watson et al., 2012). Moreover, programming error messages are sometimes imprecise: (1) the same error in code may produce different error messages in different context; and (2) the same error message may result from different errors in code (McCall and Kölling, 2019). Thus, the ambiguous and imprecise error messages become a significant barrier to students’ success in introductory programming (Stefik and Siebert, 2013; Becker, 2016).

To help students better understand the cryptic programming error message, researchers started to explore error message enhancement, and early work dates back to 1960s. In 1965, researchers at Purdue University developed a translator for FORTRAN called PUFFT (Purdue University Fast FORTRAN Translator), which provided elaborate diagnostic messages (Rosen et al., 1965). In 1995, Schorsch (1995) introduced the Code Analyzer for Pascal (CAP) tool, which provided user-friendly error messages. Instead of describing syntax errors from the compiler perspective, the error messages of CAP explained syntax errors in a way that was friendly to student programmers and offered guidance on debugging code (Schorsch, 1995). Gauntlet was another tool designed to support teaching introductory programming, which explained the top fifty Java programming errors using plain language with some humor (Flowers et al., 2004). While studies on CAP and Gauntlet reported positive effects of the error message enhancement, their conclusions were based on anecdotal evidence.

Most empirical studies on programming error message enhancement were conducted after 2010, and conflicting results have been reported (Becker et al., 2019). Denny et al. (2014) examined the effects of enhanced feedback in the tool CodeWrite and did not find any significant effects. Becker (2016) designed the tool Decaf with 30 enhanced Java compiler error messages and reported significant effects of reducing student errors. Pettit et al. (2017) used historical data in the AAT Athene to design elaborate feedback for the most frequent compilation errors and examined its effects. In the study, they first analyzed Athene’s historical data of four semesters and identified the top compilation errors in the introductory C + + programming course. Next, they added enhanced compiler error messages for Athene and analyzed the student data of another four semesters. However, they did not find significant effects of the enhancements. Qian and Lehman (2019) investigated the effects of enhanced feedback messages in an introductory Java programming course for high-ability high school students. They also identified the common errors and elaborated on the original error messages. Different from previous studies focusing on reducing the number of errors, they examined students’ rates of improving erroneous programs. Given the enhanced error messages, if students can better improve their code than those receiving original error messages, it indicates the enhanced error messages work. Significant effects were found in their study (Qian and Lehman, 2019).

### Purpose of the Study

Existing research mainly focuses on compiled programming languages such as Java and C + +. Few studies have examined error message enhancement in interpreted programming languages such as Python. Moreover, most studies investigate college students using AATs with enhanced programming error messages; few have studied younger students such as middle or high school students. Finally, although early work on error message enhancement dates back to 1960s, its effectiveness is still inconclusive (Pettit et al., 2017; Becker et al., 2019). The goal of this study was to explore the effectiveness of enhanced programming error messages (EPEMs) in a Python-based introductory programming course. Different from previous studies, our study investigated the effects of EPEMs on middle school students learning introductory programming. Furthermore, previous research only examined the effects of EPEMs from the angle of error frequencies, but our study analyzed more comprehensive data including students’ error frequencies, debugging performance, and overall learning performance. Participants of this study were two groups of middle school students. Group A was the control group only receiving raw programming error messages (RPEMs) given by the Python interpreter, and group B as the treatment group received EPEMs. We used student data in an AAT called Mulberry to explore the effectiveness of EPEMs. The following three research questions guided this study:

RQ1 Do students receiving EPEMs make fewer errors than those receiving RPEMs?
RQ2 Can students receiving EPEMs better debug their code than those receiving RPEMs?
RQ3 Do students receiving EPEMs show better performance in introductory Python programming than those receiving RPEMs?

## Materials and Methods

### Participants and Context

The participants of this study were two groups of 7th graders from a public middle school in China. This school had about 450 students, and most students were from middle class families. In China, middle schools have three grades: 7, 8, and 9th grade. This school had four classes in each grade. We randomly chose two classes in 7th grade, designated group A and group B, as our research groups. Group A was the control group that received RPEMs while group B was the treatment group that received EPEMs.

Originally, both groups had 36 students. However, one student of group A and three students of group B were absent for several class sessions and did not take the final programming exam. Hence, we excluded them from our data analysis. Thus, we had 35 students in group A and 33 students in group B. According to the data given by the assessment department of the school, students of the two groups were similar in age and performance in core academic subjects including Chinese, Math, and English (see Table 1). In other words, students of the two groups had similar cognitive abilities.

**TABLE 1:** Academic scores of core subjects and age.

	N	Chinese *Mean (SD)*	Math *Mean (SD)*	English *Mean (SD)*	Age *Mean (SD)*
Group A	35	78.40 (4.73)	87.80 (7.86)	89.99 (3.55)	12.49 (0.38)
Group B	33	80.64 (5.12)	87.30 (6.54)	91.62 (3.70)	12.58 (0.34)

Students of the two groups took the same introductory programming course called *Introduction to Python programming* in Fall 2020. They attended a 90-minute course block every week for 14 weeks. Python programming topics covered in the course included Input and Output (I/O), Variables, Operators, Conditionals, and Loops. The Python version was 3.7, and the coding environment was Mu,^1^ which is a code editor designed for Python learners.

The automated assessment tool (AAT) used in this course was Mulberry. It had 64 programming problems related to different topics covered in the course. Students had to write programs from scratch to solve problems in Mulberry. When they made errors in code, Mulberry would provide error messages for them. According to the error messages, students could revise and improve their erroneous code and resubmit their solutions until their solutions were correct. For more details about Mulberry, please see Qian and Lehman (2021).

### Procedures

According to previous studies, the first step for designing EPEMs is to identify common language specification errors (LSEs). According to Qian and Lehman (2020), common LSEs as errors made by at least a third of the students. We used their standard to identify common errors. The data used for the identification of common LSEs was from our pilot study in 2019. In the pilot study, a group of 35 students in the 7th grade of this middle school took the introductory Python programming course. Based on their data and the identification standards mentioned above, eight common LSEs were identified (Appendix A presents details of the eight LSEs).

**FIGURE 1:**
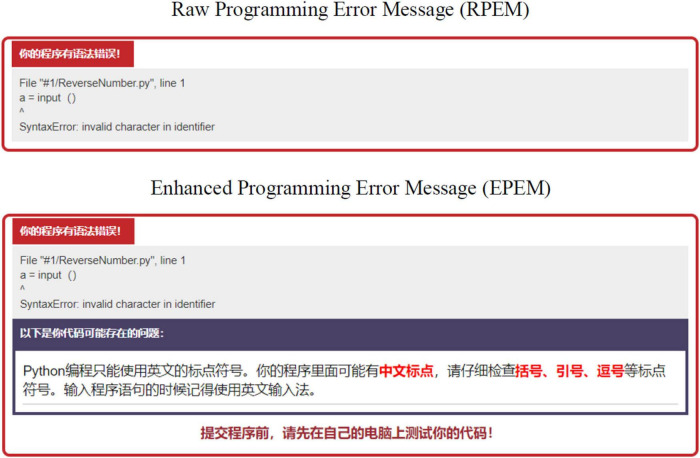
Screen Shots of the RPEM and EPEM for “invalid character in identifier”.

After analyzing students’ erroneous code related to the eight common errors, EPEMs were designed. For instance, using Chinese punctuation marks in Python code was one of the common LSEs and led to the error message “invalid character in identifier.” The raw programming error message (RPEM) was not informative. For the EPEM, we added explanation about the error in the code and included possible directions of debugging. Figure 1 presents the screen shots of the RPEM and EPEM for this error. Appendix A presents details about the common LSEs and the translated EPEMs^2^.

When students of the two groups used Mulberry to solve problems, they received different feedback when encountering a common LSE. For group A, they received RPEMs. For group B, they received EPEMs. Mulberry automatically collected all the student solutions. The erroneous ones were data source for analyzing students’ error frequencies and common errors. The improvements in students’ solutions to a certain problem were data source for analyzing students’ performance of debugging code. The experiment lasted 14 weeks in Fall 2020. At the end of the semester, we exported the data from Mulberry for data analysis.

### Data Analysis

To answer RQ1, the number of students’ LSEs was analyzed and compared to see whether group B (EPEM group) made fewer LSEs than group A (RPEM group). As EPEMs were only provided for the eight common LSEs, we also analyzed the number of occurrences of the eight common LSEs.

To answer RQ2, we analyzed and compared students’ performance of debugging code. Previous studies have developed measures describing students’ debugging performance, including Error Quotient (Jadud, 2006) and Improvement Rate (Qian and Lehman, 2020). Error Quotient (EQ) is a number between 0 and 1 describing students’ ability to fix LSEs. Students with lower EQs are better able to fix LSEs. See Jadud (2006) for details about the calculation of EQ. Improvement Rate (IR) can be used to describe both students and LSEs. For a specific LSE, if it occurs in solution N and gets fixed in solution N + 1, it means that the erroneous solution gets improved, and the proportion of the improved solutions is the IR of the LSE. For a student, the proportion of the improved solutions is his or her IR. See Qian and Lehman (2020) for details about the calculation of IR. By using the two indicators, we first compared the two groups’ IRs of the eight LSEs with EPEMs. Next, we compared students’ EQs and IRs to see whether group B showed a better performance.

To answer RQ3, we compared students’ learning performance in the introductory programming course. At the end of the semester, a final programming exam was given to the students. In the final exam, students had to write short programs from scratch to solve problems. The exam scores were used to compare their learning performance in the course.

## Results

### Enhanced Programming Error Messages Was Not Effective in Reducing Students’ Errors

Students of group A submitted 3391 solutions in total, of which 788 had LSEs. Students of group B submitted 2948 solutions in total, of which 799 had LSEs. Table 2 presents details about the number of solutions and errors of the two groups. The proportion of LSEs of group B (27.1%) was higher than it of group A (23.2%), *z* = 3.54, *p* < 0.01. On average, students of group B made about 1.7 more LSEs than students of group A did. It is surprising that students receiving EPEMs made LSEs more frequently than those receiving RPEMs. In other words, EPEMs did not help to reduce students’ LSEs.

**TABLE 2:** Comparison of two groups’ LSEs.

	*N*	Total solutions	Number of LSEs	Percentage of LSEs	LSEs per student
Group A	35	3391	788	23.2%	22.51
Group B	33	2948	799	27.1%	24.21

According to the analysis of the occurrences of the common LSEs, we found that most of the common LSEs were similar between the two groups (see Table 3). For group A, LSE 7 and 8 were not identified as common according to our standards, but for group B, LSE 7 was a common error. For both groups, common LSEs accounted for about 90% of all LSEs.

**TABLE 3:** Comparison of two groups’ common LSEs.

#	Error	Group A		Group B	
			
		Students	Frequency	Students	Frequency
LSE1	Invalid syntax	33/35	258 (32.7%)	31/33	267 (33.4%)
LSE2	NameError	29/35	103 (13.1%)	29/33	163 (20.4%)
LSE3	ValueError	28/35	101 (12.8%)	25/33	64 (8.0%)
LSE4	TypeError	23/35	114 (14.5%)	24/33	107 (13.4%)
LSE5	IndentationError	20/35	75 (9.5%)	16/33	46 (5.8%)
LSE6	EOFError	12/35	58 (7.4%)	17/33	35 (4.4%)
LSE7	invalid character in identifier	9/35	27 (3.4%)	16/33	41 (5.1%)
LSE8	EOL while scanning string literal	6/35	18 (2.3%)	7/33	11 (1.4%)

### Enhanced Programming Error Messages Was Not Effective in Helping Students Better Debug Code

Although our results did not indicate positive effects of enhanced error messages on reducing students’ LSEs, it may help students better debug erroneous programs, as enhanced error messages were designed for this purpose. Unfortunately, we did not find such positive effects in our data either.

**FIGURE 2:**
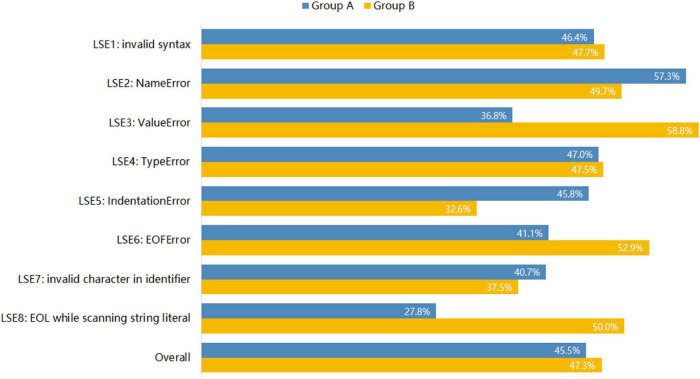
Improvement Rates of LSEs with EPEMs.

Firstly, we analyzed and compared improvement rates (IRs) of the eight LSEs with EPEMs. Figure 2 presents the IRs of each LSE and the overall IR of all the LSEs. The overall IRs of the two groups were very close, 45.5% for group A and 47.3% for group B. In other words, EPEMs did not help students fix the relevant errors and improve their code. For LSE2 and LSE7, group A even showed higher IRs than group B, although the differences were not statistically significant. For LSE8, group B seemed to perform much better than group A on fixing the error, but LSE8 occurred rarely for both groups and was not identified as a common error for both groups. Further, the difference was not significant. Only for LSE3, group B (58.8%) showed a significant higher IR than group A (36.8%), *z* = 2.20, *p* < 0.05. This error mainly resulted from incorrectly using *int()* function to convert input data which were not integers (e.g., “3.14”). In general, EPEMs did not show significant effects on helping students rectify relevant errors.

Second, we analyzed and compared students’ performance on debugging code using the indicators IR and EQ. As EPEMs were only provided for the eight common LSEs, we also calculated and compared students’ IRs and EQs specifically for those eight LSEs (see IR of EPEMs and EQ of EPEMs in Table 4). For all the indicators, no significant differences were found. In other words, students receiving EPEMS showed similar performance in debugging with those receiving RPEMS.

**TABLE 4:** Comparisons between two groups in IR and EQ.

	Group A (*N* = 35)		Group B (*N* = 33)			
		
	*Mean*	*SD*	*Mean*	*SD*	*t*	*p*
IR	0.77	0.14	0.73	0.13	1.27	0.21
IR of EPEMs	0.59	0.22	0.54	0.19	0.97	0.33
EQ	0.16	0.14	0.19	0.11	−0.97	0.33
EQ of EPEMs	0.39	0.21	0.42	0.18	−0.66	0.5

### Students Receiving Enhanced Programming Error Messages Did Not Show Better Learning Performance Than Those Receiving Raw Programming Error Messages

Enhanced programming error messages also did not show any positive effects on students’ learning performance in introductory programming. The final exam scores of the two groups were very close (see Table 5). As group B did not show superiority in any indicators in previous two sections, it is not surprising that they showed similar learning performance as group A. In this study, EPEMs appear to be ineffectual in helping students debug erroneous code and improve their performance in introductory Python programming.

**TABLE 5:** Comparisons between two groups in learning performance.

	*N*	Mean	SD	Median	Min	Max
Group A	35	69.46	10.95	71	49	90
Group B	33	69.52	9.16	71	50	88

## Discussion

### Effectiveness of Enhanced Programming Error Messages

According to our results in this study, programming error message enhancement did not help to reduce student errors or improve students’ performance in debugging. Students of group B who received EPEMs even made more LSEs than students of group A who received RPEMs. For other measures such as IR, EQ, and overall learning performance, students of the two groups were similar to each other. Our results are consistent with prior studies on college students learning Java (Denny et al., 2014) and C + + (Pettit et al., 2017). As programming error messages do not always report the actual error in code (Kohn, 2019; McCall and Kölling, 2019), it is possible that the EPEMs designed based on the error messages provided by the Python interpreter did not offer useful explanations for the actual error in student code. For example, *LSE1: invalid syntax* in Python may result from many different mistakes in code, such as failing to put strings within quotation marks, incorrectly using operators, and so forth. As the RPEM does not provide much information about the actual error in code, the EPEM is not very informative either. Thus, it may be ineffective to help students debug code.

In addition, because our participants were two groups of Chinese middle school students, difficulties in typing code and their stage of cognitive development may also influence the effectiveness of EPEMs. Previous research has found that typing code is a challenge for Chinese middle school students (Qian and Lehman, 2021). On the one hand, middle school students are young and may not have fluent keyboarding skills. On the other hand, Chinese students may mistakenly use Chinese punctuation marks in code, which are invalid in Python programming. When students struggle with typing code and also try to fix an erroneous program, they may not pay enough attention to the EPEMs, as several challenges occur simultaneously. Meanwhile, according to Piaget’s stages of cognitive development, middle school students are not cognitively mature as college students and usually are still developing from concrete to formal operational thought (Huitt and Hummel, 2003). They may have more difficulties in understanding certain programming concepts than older students do. Thus, some explanation provided by the EPEMs may not be meaningful to middle school students.

While some previous studies reported positive effects of EPEMs (Becker, 2016; Qian and Lehman, 2019), their analysis only focused on specific dimensions of data and did not examine the effects of EPEMs on students’ learning performance in introductory programming. For instance, Becker (2016) found that enhanced compiler error messages reduced the occurrences of common Java errors and repetition of the errors. However, his study did not indicate a positive relationship between EPEMs and enhanced learning performance. Making fewer errors does not necessarily result in better performance in learning. In our study, group A made fewer errors than group B, but their learning performance did not show any difference. Qian and Lehman (2019) reported that EPEMs led to higher IRs in introductory Java programming, but again they did not investigate whether there existed a positive relationship between EPEMs and learning performance. As the purpose of designing EPEMs is to help students better learn programming, some positive effects may not be important if EPEMs do not improve students’ learning performance.

### The Viewpoint of Productive Failure

In our study, EPEMs appeared to be ineffectual to help students in introductory programming. Our findings also seem to be consistent with previous research on EPEMs. However, rather than demonstrating the ineffectiveness of EPEMs, we believe that there could be another explanation: students learn from their failures. In other words, the cryptic and confusing raw errors messages (Becker et al., 2019) may promote learning in certain way. Some difficulties during learning seem to hinder short-term performance but may lead to better learning in the longer term (Schmidt and Bjork, 1992). Thus, debugging code provides opportunities for *productive failure* (Kapur, 2016) or the “failure precedes later success in learning” (Kafai et al., 2019, p. 169). In our study, receiving RPEMs might be perceived as a disadvantage to group A, but making mistakes and fixing code with only RPEMs may help students learn in a deeper way, especially in conceptual knowledge and transfer, according to the viewpoint of productive failure (Kapur, 2008, 2016). For instance, when students made *LSE5: IndentationError* in code receiving only the RPEM, they had to understand the special meaning of indentation in Python so that they could fix the error. For the students receiving the EPEM (see Appendix A), they just needed to find the extra spaces or missing colons to fix the error. In such situation, understanding the true meaning of indentation in Python was not necessary, and they might repeat the error next time. Hence, although RPEMs are cryptic to novices and do not contain much information for debugging code (Becker et al., 2019), the process of understanding RPEMs and successfully fixing the errors provides extra opportunities for students to learn programming.

Therefore, it is not so surprising that prior studies and our results both found ineffectiveness of EPEMs. For a single error in code, EPEMs may have some positive effects in a short term. However, for the whole semester, we should not ignore the positive effects of handling errors without EPEMs. Debugging itself is an integral part of learning to program. Kafai et al. (2019) suggest “rethinking debugging as productive failure for CS education” (p. 169). From this perspective, we recommend that researchers of EPEMs should reconsider the role of error messages and redesign research and instruction.

### Limitations and Future Research Directions

Our study has several limitations. First, compared to previous studies, our dataset was relatively small. We analyzed about 6,300 student solutions. For instance, Pettit et al. (2017) examined 36,050 student solutions over eight semesters. In addition, our group size was also relatively small. In the study of Becker (2016), he had about 100 students in each group. We believe that larger dataset and sample size could improve the generalizability of findings. Third, we did not administer a pre-test for measuring students’ existing programming knowledge, which could have certain effects on the results. Finally, as programming error messages do not always report the actual error in code, our design of the enhanced error messages may not accurately explain the actual error. Hence, future research is needed to find an approach to identify the actual error in students’ erroneous code.

Our results also provide potential directions for future studies on EPEMs. First, according to the viewpoint of productive failure, future research should pay attention to students’ debugging process and investigate whether and how failures can be productive. In addition, as AATs collect comprehensive data of students’ learning progress, it is vital to use such data to examine students’ learning and identify important predictors of students’ success in introductory programming.

## Conclusion

This study explored the effectiveness of EPEMs in a Python-based introductory programming course using an AAT called Mulberry. After analyzing the data of two groups of middle school students, we found that EPEMs did not help to reduce student errors or improve students’ performance in debugging. The EPEM group even made more errors than the RPEM group. For other measures such as IR, EQ, and overall learning performance, students of the two groups were similar to each other. Our results are consistent with prior studies on EPEMs. The ineffectiveness of EPEMs may result from reasons such as the inaccuracy of the interpreter’s error messages or students not reading the EPEMs. However, the viewpoint of productive failure may provide a better explanation of the ineffectiveness of EPEMs. While the raw error messages are cryptic and confusing to students, the difficulties and failures can be resources for learning. Students who struggle to understand typical error messages may learn more in the long run. Hence, we recommend that researchers reconsider the role of errors in code and investigate whether and how failures and debugging contribute to the learning of programming.

## Data Availability Statement

The raw data supporting the conclusions of this article will be made available by the authors, without undue reservation.

## Ethics Statement

Ethical review and approval was not required for the study on human participants in accordance with the local legislation and institutional requirements. Written informed consent from the participants’ legal guardian/next of kin was not required to participate in this study in accordance with the national legislation and the institutional requirements.

## Author Contributions

ZZ collected and analyzed the data and drafted the manuscript. SW reviewed the literature and proposed the research design. YQ designed the EPEMs and helped to draft the manuscript. All authors contributed to the article and approved the submitted version.

## Conflict of Interest

The authors declare that the research was conducted in the absence of any commercial or financial relationships that could be construed as a potential conflict of interest.

## Publisher’s Note

All claims expressed in this article are solely those of the authors and do not necessarily represent those of their affiliated organizations, or those of the publisher, the editors and the reviewers. Any product that may be evaluated in this article, or claim that may be made by its manufacturer, is not guaranteed or endorsed by the publisher.

## Footnotes

1
codewith.mu/

2 Students saw Chinese EPEMs when using Mulberry system.

## Appendix

**Appendix A:** Common LSEs and the translated EPEMs.

#	Errors	Translated enhanced programming error messages (EPEMs)
LSE1	Invalid syntax	Your program has statements that do not follow the Python syntax. For example, failing to put strings within quotation marks, incorrectly using operators, and so forth. Please carefully read your program again. Note: Python does not have any intelligence and can only understand code that meets its rules.
LSE2	NameError	Probably, you used wrong variable or function names in your code. Please check if you misspelled any variable or function names. It is also possible that you used a variable in expressions before initialization.
LSE3	ValueError	Your program had something wrong the value of variables. You probably used the *int()* function to convert a variable or the input data into the *int* type. Please make sure that the value given to the *int()* function is able to be converted into the *int* type. For example, you may have wrong code like *int(′′3.14′′)*. Please read the problem description carefully, particularly the example test cases.
LSE4	TypeError	This error is about the type of the variables and related operations. First, please check if you forgot to add parentheses after the *input* (e.g., *a* = *input*). Second, the *input()* function reads data as strings (the *str* type), and you must do type conversion before any arithmetic operations. You may have wrong operations like *str * str*, *int* + *str*, and so on.
LSE5	IndentationError	You did not correctly indent your code. You may have extra spaces at the beginning of a line. Please also check the conditionals or loops to see whether you missed the colon or failed to use the same level of indentation in the code below the colon.
LSE6	EOFError	Please read the problem description carefully and pay attention to the input data part. Your program has to read the correct number of input data by using the *input()* function. It is possible that your program used the *input()* function too many times when the given input data was only one number or a string. Please read the problem description carefully, particularly the example test cases.
LSE7	Invalid character in identifier	You used Chinese punctuation marks in your code! Python only accepts English punctuation marks. Please read your code carefully to see if you used any Chinese parentheses, quotation marks, commas, and so on. Do not forget to use English input mode when typing code.
LSE8	EOL while scanning string literal	You may have mismatched or missing quotation marks. Make sure you have them in pairs.
